# Contextual factors favouring success in the accreditation process in Colombian hospitals: a nationwide observational study

**DOI:** 10.1186/s12913-020-05582-y

**Published:** 2020-08-20

**Authors:** Mario A. Zapata-Vanegas, Pedro J. Saturno-Hernández

**Affiliations:** 1 CES University, School of Medicine, Medellin, Colombia; 2 National Institute of Public Health, Av. Universidad 655, CP62100 Cuernavaca, Morelos Mexico

**Keywords:** Quality improvement, Hospital accreditation, Context

## Abstract

**Background:**

To identify context factors associated with and predicting success in the hospital accreditation process, and to contribute to the understanding of the relative relevance of context factors and their organizational level in the success of QI initiatives.

**Methods:**

Analytical study of cases and controls in a sample of hospitals of medium and high complexity in Colombia. Cases (*n* = 16) are accredited hospitals by the time of preparation of the study (2016) and controls (*n* = 38) are similar facilities, which have not succeeded to obtain accreditation. Eligibility criteria for both groups included complexity (medium and high), having emergency services, an official quality assurance license, and being in operation for at least 15 years. Besides eligibility criteria, geographical location, and type of ownership (public/private) are used to select controls to match cases.

Context measures are assessed using a survey instrument based on the MUSIQ model (*“Model for Understanding Success in Quality”*) adapted and tested in Colombia. Statistical analysis includes descriptive measures for twenty-three context factors, testing for significant statistical differences between accredited and non-accredited hospitals, and assessing the influence and strength of association of context factors on the probability of success in the accreditation process. A multivariate model assesses the predictive probability of achieving accreditation.

**Results:**

Eighteen (78.3%) of the twenty-three context factors are significantly different when comparing cases and controls hospitals, particularly at the *Microsystem* level; all factors are statistically significant in favor of accredited hospitals. Five context factors are strongly associated to the achievement of accreditation but in the logistic multivariable model, only two of them remain with significant OR, one in the *Macrosystem,* “Availability of economic resources for QI” (OR: 22.1, p: 0,005), and the other in the *Microsystem*, “Involvement of physicians” (OR: 4.9, p: 0,04).

**Conclusion:**

This study has applied an instrument, based on the MUSIQ model, which allows assessing the relevance of different context factors and their organizational level in hospitals, to explain success in the accreditation process in Colombia. Internal *macrosystem* and *microsystem* seem to be more relevant than external environment factors.

## Background

There are currently a broad array of methods, techniques and interventions that may improve performance and quality in healthcare organizations [1–3]; however, there is still little evidence in relation to the influence of context and its identifiable factors which may facilitate improvement [4–6]. Some studies have attempted to signal context predictors that can lead to successful implementation of quality improvement (QI) initiatives [7, 8], but this issue remains a relevant subject for research and policy making in the quality improvement field.

*Context* has been defined within the multi-level organizational theory as *“the whole structure of connections between the components, which provide meaning to these components”* [9]. This definition suggests the importance of evaluating health organizations as a whole and the need to recognize not only the contribution of individual elements and factors, but also the contribution of the interaction between these to the outcomes of healthcare organizations.

In the last decade, authors [10, 11] have agreed on the need for research that deepen in the understanding of the role of context in quality improvement and the implementation of effective interventions that improve outcomes in healthcare. A noteworthy contribution in this search is the MUSIQ model [11] (*The Model for Understanding Success in Quality*) proposed by Kaplan and colleagues [12] after a systematic review. They concluded that research and knowledge on the subject is in an early stage of development and observed more than sixty-six elements and factors that may be relevant to QI, but without a definitive conclusion about the weight that each one of them may have for success. This review gave foundation to the proposed model, which in its original form identified twenty-five factors that may influence success in the implementation of QI projects. These factors are grouped by organizational level according to the place of interaction. In this way, the MUSIQ model includes features of the external *Ambient*, of the organization as a whole, that configures the *Macrosystem,* and the *Microsystem*, which refers to the operation of its services providing care to patients. The model also includes the interrelation with the manager and the team for Q I[11, 13]. The MUSIQ model has been used in a number of countries [6], but to our knowledge it has not been adapted and applied neither in the Latin-american environment nor having accreditation as the objective of a quality improvement initiative.

In this study, we used the MUSIQ model, previously adapted and pilot tested in Colombia, to assess the influence of context factors in the achievement of accreditation in medium and high complexity hospitals, comparing accredited hospitals to those have not succeeded. Accreditation is considered as a dynamic strategy that can contribute to the improvement of processes and results in health [14–16], and a desirable, but voluntary, status in the Colombian health system. Accreditation started in Colombia in 2002 as part of the implementation of the, so-called, comprehensive system of quality assurance in health, and after a thorough reform of the health sector during the 90’s decade. It is now included in the official *Sistema Obligatorio de Garantía de Calidad* (SOGC: Mandatory System for Quality Assurance), which also encompasses licensing of facilities and quality audits. Accreditation is regarded as the maximum achievement of quality standards, but it is not related to payment incentives but rather to the potential prestige of the institution. In this study, we intend to identify those context factors associated with and predicting success in the accreditation process in Colombia, and to contribute to the understanding of the relative relevance of the different context factors and their organizational level in the success of QI initiatives.

## Methods

### Population and sample

We performed an analytical study of cases and controls, where cases are the accredited hospitals and the controls are similar facilities, which have not succeeded to obtain accreditation. Both groups were recruited by non-probabilistic consecutive cases sampling method, from the total population of public and private hospitals of medium and high complexity in Colombia. The level of complexity is officially determined and relates to the type of services offered by the hospital. A medium complexity hospital will have at least general surgery and internal medicine, and support services such as clinical laboratory and radiology; a high complexity hospital will have Intensive Care Unit as well as other medical and surgical specialties and sub-specialities.

As for the date of the preparation of this study (2016), thirty-two health facilities were accredited in Colombia, and twenty-three of them were eligible (eligibility criteria are described below) for our study. The location and characteristics of eligible accredited hospitals were used to select matched controls through the official register of providers of healthcare services (REPS) [17]. The parameters to determine the desirable sample size for the study were a confidence level of 95%, power of 80%, with a case-control ratio of 1:3, and exposure factor to test (“favorable context conditions for the accreditation of cases”) 90% vs. 50% in control hospitals. These parameters required a sample size of 56 hospitals, 14 *cases* (accredited) and 42 *controls* (non-accredited), of a total population of eligible 23 and 131 respectively.

The general eligibility criteria for both cases and controls were, complexity for health problems resolution (medium and high complexity hospitals, according to infrastructure and type of services provided), with both emergency and in-patient services, licensed by the official system of quality assurance (SOGC) [18] and that have been in operation for at least 15 years. The specific eligibility criteria were to be accredited by the external accreditation body at the time of the study (cases) [19], or, for controls, have either ongoing or developed initiatives in the past 10 years aimed to obtain accreditation, but had not achieved it [20, 21]. The geographical location in the same city or region of influence, the type of ownership (public or private), and the similarity in the level of complexity (infrastructure, type, quantity and diversity of specialists and departments for the provision of in-patient care), are used to select *controls* with reference to the *cases.*

The twenty-three eligible accredited hospitals were invited to participate in the study via e-mail, with an average of six e-mail reminders, reinforced by telephone contact (three calls on average), during a defined period of 8 months. A similar approach was undertaken to recruit controls, targeting the total eligible hospitals. Eventually, after the 8 months recruitment period, 16 accredited hospitals (two more than the required sample) and 38 non-accredited (four less that the expected sample) participated in the study. Overall, the planned power and confidence was achieved. A table describing the frequency of matching variables (ownership and complexity) in cases and controls hospitals, as well as the type of policy for human resources recruitment, is included as supplementary material (Annex 1).

### Instrument for measuring context elements and factors

We used a survey instrument based on the MUSIQ model (*“Model for Understanding Success in Quality”*) [11, 13], translated into Spanish and adapted and tested in Colombia for psychometric validity [22] with authorization of its lead author (H. Kaplan). The adapted instrument [23], consists of 35 items grouped into 23 factors (some of them single-item, and some others incorporating previously validated scales) to characterize four levels of context (Environment, Macrosystem, Microsystem, and QI team) Table 1. The Environment is external to the facility, while the Macrosystem and Microsystem levels are internal and provide structure and functionality for the operation and provision of services; and the QI team is formed by those leading or in charge of a quality improvement project. Answers to the instrument have the format of a Likert-type [28] 5-points (choices of response) rating scale. The questions explored in the instrument, grouped by context levels, are described in as supplementary material (Annex 2).

**Table 1:** Dimensions, factors and items to assess context for success in the accreditation process (adapted from the MUSIQ [9] model)

Context Dimension	Context Factors (variables)	Item #
Environment	External incentives/market	1
	Board of Trustees/ directors strategic decision	2
	External sponsorship for the development QI projects	3
	Institutional prestige	4
Macrosystem	Management leadership for QI at the hospital	5
	Existence of Director or Manager for quality	6
	Culture for QI in the organization [24]	7–10
	Organizational maturity for QI	11
	Priority for development of human talent for QI [25]	12–15
	Availability of resources for QI	16
	Information systems support for QI	17
	Employment stability	18
Microsystem	Leadership for QI in the Microsystem [26]	19,20,23
	Motivation for QI	21
	Skills for utilization of tools for QI	22
	Culture for QI in the Microsystem [24]	23–25
	Involvement of physicians	26
Quality Improvement team	Leadership for managing quality	27
	Diversity of the quality team	28
	Experience and specific knowledge on QI	29
	Teamwork [27]	30–33
	Skills in procedures and techniques for QI	34
	Stability of the quality team	35

*MUSIQ* Model for Understanding Success in Quality, *QI* Quality Improvement

### Data gathering and statistical analysis

Hospital complexity (infrastructure and type of specialized services provided), the type of ownership (public or private), number of employees and forms of recruitment were used to match cases and controls, and used for multivariable analysis as explained below.

For the administration of the survey to gather context data, we developed a web-based, self-administered questionnaire addressed to the Medical Director and the Director of Quality of the surveyed hospitals. The resulting information was exported to SPSS*® Statistics 21 version (license CES University-Colombia).*

The statistical analysis plan was two-fold. First, we estimate central tendency (mean, median) and dispersion (standard deviation, interquartile range) measures for the 23 factors, and tested for significant statistical differences between accredited and non-accredited hospitals, using Student’s t test when data were normally distributed (Shapiro-Wilk test) or, otherwise, the Mann-Whitney test. For the only one categorical (binomial) variable in the survey, we test differences using the chi-square approach.

Second, to look at the influence and strength of association of context factors on the probability of success in the accreditation process, we estimate the Odds Ratio (OR) with 95% confidence interval. This estimation is made first for every single context factor, and then altogether using a logistic multivariable model, where being accredited (yes/no) is the dependent variable and the context factors and structural data the independent variables, entering in the model with step forward method [29] and using all the factors. For this analysis we dichotomize the 5-options scale answers, which is convenient for clarity in the interpretation of Odds Ratios (OR) and also because some values could be 0 (zero) and the OR could not be calculated. We considered values between 1 and 3 as a negative exposure (i.e. disagreement with the statement of progress in the development of the context element), and values greater than 3 are interpreted as a positive exposure (agreement with the statement of progress in the development of the context element).

The logistic multivariate model was used also to determine possible predictor variables, their combination and contribution to the predictive probability of achieving accreditation, adjusting by the effect of the other context variables.

## Results

### Differences in context factors between accredited and non-accredited hospitals

Because of the matching design of the sample, there were no significant differences in level of complexity, size and type of ownership between cases (success in the accreditation process) and controls (have not succeeded to obtain accreditation). However, assessment of eighteen (78.3%) of the twenty-three context factors are significantly different when comparing cases and controls hospitals. These differences occur in all the levels, but particularly at the *Microsystem*, where all its five factors are statistically significant in favor of accredited hospitals (Table 2). In this level, the “Active involvement of physicians” is valued much higher in accredited hospitals (median = 4.0 vs. 2.0). Next level where the majority of factors (seven out of eight) are significantly different in favor of accredited hospitals is the “*Macrosystem*”. The only *Macrosystem* factor equally valued in both groups is the “Existence of Quality Manager or Director”, indicating that this position exists in all the assessed hospitals.

**Table 2:** Comparative assessment of context factors in accredited (cases) vs. non-accredited (controls) hospitals

Context Factor (variable)	Cases		Controls		***P***-value
	Median or Average	IR or SD	Median or Average	IR or SD	
**Environment**					
External incentives/market ^a^	2.00	2.50	3.50	2.00	**0,03**
Board of Trustees/ directors strategic decision ^c^	100%		73.7%		**0,02**
External sponsorship for QI projects ^a^	1.00	2.50	1.00	2.00	0,96
Institutional prestige ^a^	4.00	2.00	4.00	1.00	0,06
**Macrosystem**					
Management leadership for QI ^a^	5.00	0.00	4.00	1.00	**0,002**
Existence of Director or quality Manager ^a^	5.00	0.00	5.00	0.00	0,34
Culture for QI in the Organization ^b^	4.33	0.47	3.70	0.77	**0,004**
Organizational maturity for QI ^a^	5.00	0.00	4.00	1.00	**0,004**
Priority for development of human talent QI ^b^	4.13	0.66	3.31	0.77	**0.0005**
Availability of resources for QI ^a^	5.00	1.00	3.00	2.00	**0.0000**
Information systems support for QI ^a^	4.00	1.00	3.00	2.00	**0,002**
Employment stability ^a^	4.00	1.50	3.00	2.00	**0,01**
**Microsystem**					
Leadership for QI in the Microsystem ^a^	4.67	0.67	4.17	1.00	**0,004**
Motivation for QI ^a^	5.00	1.00	4.00	0.00	**0,0009**
Skills for utilization of tools for QI ^a^	5.00	1.00	4.00	2.00	**0.0002**
Culture for QI in the Microsystem ^b^	4.33	1.00	4.00	1.00	**0,0004**
Involvement of physicians ^a^	4.00	2.50	2.00	1.00	**0,0025**
**Quality Improvement team**					
Leadership for managing quality ^a^	5.00	0.00	5.00	1.00	**0,015**
Diversity of quality team ^a^	5.00	0.00	4.00	1.00	**0,009**
Experience and specific knowledge on QI ^a^	5.00	1.00	5.00	1.00	0,37
Teamwork ^a^	4,75	0,38	4,50	0,75	**0,014**
Skills in techniques and procedures for QI ^a^	5.00	0.00	5.00	1.00	0,68
Stability of the quality team ^a^	5.00	1.00	4.00	1.00	**0,003**

^a^ not normally distributed variables. Central tendency measure: Median; Dispersion measure: Interquartile range (IR); Statistical test: Mann Whitney U

^b^normally distributed variable. Central tendency measure: Mean; Dispersion measure: Standard Deviation (SD); statistical test: Student’s t

^c^ categorical (binomial) variable. Statistical test: Chi-square

At the “*Quality Improvement team*” level, two seemingly relevant factors showed no significant differences between accredited and non-accredited hospitals. These are: 1) “QI experience and specific knowledge of the QI team”; and 2) “skills of the QI team for the use of techniques and methods for QI”; even though the dispersion of data for the latter is smaller in the group of accredited hospitals. The other four factors included in this dimension are significantly better in the accredited hospitals, including “Leadership for managing quality”, “Diversity of quality team”, “Team work” and “Stability of the QI team”.

In the *“Environment”* level**,** the one including factors outside or beyond the facilities themselves, two of the four factors show significant differences. One of them (“*External market-driven motivations*” is felt greater in non-accredited hospitals (median: 3.5 vs 2.0 in accredited facilities, p: 0,03); while the other (“*Strategic decision of the Board of Trustees or Directors*” for the achievement of accreditation) is present significantly more in the accredited facilities (100% vs 73.7%, p:0,02). The search for *“Institutional prestige”* seems to be equally important for both groups (median 4.0 in both), but more consistently (less dispersion of values) for the non-accredited.

### Context factors significantly associated to the probability of success in the accreditation process

Analyzing them one by one, five of the twenty-three context factors are strongly associated to the achievement of accreditation (Table 2). All of them are included in the context levels within the facilities. They are the following, ordered by the strength of the association, estimated by the Odds Ratio (OR) for the exposure to a given context factor: 1. *“Availability of economic resources to the MC”,* in the Macrosystem (OR = 28.9); 2*. ‘Capabilities for the use of tools for the QI’* in the Microsystem (OR = 13.5); 3.*“Supporting information for QI systems”* in the Macrosystem (OR = 7.8); 4*. “Stability of the quality team”* (OR = 7.8); and 5*. ‘Involvement of physicians’* in the Microsystem (OR = 7.4).

In the logistic multivariable model, only two of them remain with significant OR, adjusting by all the other context factors (Table 3). One in the *Macrosystem*: *“Availability of economic resources for QI” (*OR = 22.1, p:0.005*)*, and the other in the *Microsystem: “Involvement of physicians”* (OR = 4.9, p:0,04*).* Using these results in a mathematical equation (eq. 1) to predict the probability for the achievement of accreditation:

1 $$ P\left(Y=1\right)=1/\Big(1+{\mathrm{e}}^{-\left[-3.66+\left(3.09\ \mathrm{availability}\ \mathrm{resources}\right)+\left(1.58\ \mathrm{involvement}\ \mathrm{of}\ \mathrm{physicians}\right)\right]} $$

**Table 3:** Dimensions and context factors significantly associated to success in the accreditation process. Single variable and multivariable logistic regression models

Single variable models				Multivariable model	
Dimension	Context factor (independent variables)	OR (95%CI)	***p*** value	OR (95%CI)	***p*** value
Macrosystem	Availability of resources for QI	28.9 (3.4–243.3)	0,0001	22.1 (2.5–194.9)	0.005
	Information systems support for QI	7.8 (1.6–39.0)	0,006	–	–
Microsystem	Skills for utilization of tools for QI	13.5 (1.6–112.7)	0,004	–	–
	Involvement of doctors	7.4 (2.0–27.2)	0,003	4.9 (1.9–21.9)	0.04
QI team	Stability of the QI team	7.8 (1.1–65.8)	0,043	–	–

*OR* Odds Ratio, *CI* Confidence interval, *QI* Quality Improvement

**Fig. 1:**
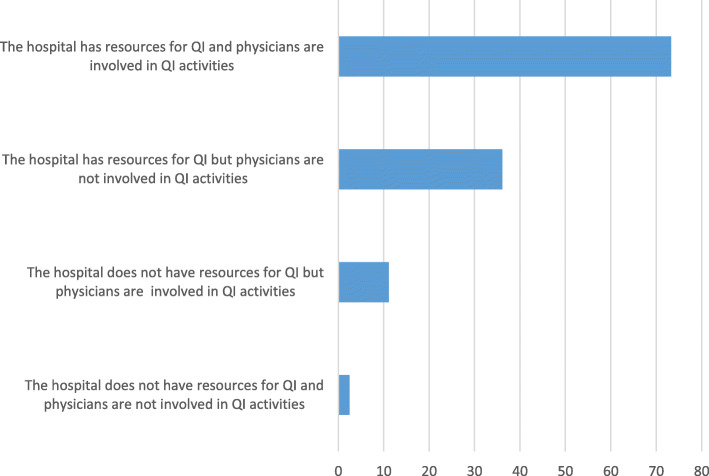
Probability of success in the accreditation process according to the presence of significant contextual factors. *Legend:* Probability of success (%) in the accreditation process according to the joint or single presence of the two significant contextual factors. Logistic model adjusted by all other context factors

The following scenarios (depicted in Fig. 1) to obtain accreditation in the sample of hospitals that participated in this study can be estimated:

- Probability of achieving accreditation if they have resources and physicians are involved in quality processes: 73.3%;
- Probability of achieving accreditation if it has resources but there is no involvement of physicians in quality processes: 36.1%;
- Probability of achieving accreditation when there are not resources for QI, but there is involvement of physicians in quality processes: 11.1%;
- Probability of achieving accreditation if they do not have resources and there is no involvement of physicians in quality processes: 2.5%.

In all cases, “not having” the context factor means that it has been assessed with values ≤3 on a five-point scale.

## Discussion

The accreditation of health care organizations is frequently recognized as a strategy that can play a relevant role for QI [15]. Some studies have explored the differences that exist in the improvement of the healthcare processes and outcomes in accredited hospitals compared with non-accredited hospitals [14, 30]; in some cases, these studies show positive results in favor of accredited facilities, even though there is yet a lack of solid conclusive evidence [31]. In other cases, the accreditation of hospitals can give response to pressures and demands of the competitive market and users, in their search for guaranteed quality health services [32, 33]. Therefore, accreditation is generally viewed as a desirable status and award, associated to quality in healthcare. Hence, the importance of understanding what context factors are most relevant to achieve accreditation, so that health care organizations could focus on them facilitating the accreditation process.

In general, the results of this study suggest that context does have an influence on the achievement of accreditation. To evaluate it, we adapted the MUSIQ conceptual model and instrument [11, 13], both to the Spanish (Colombian) language and context, and the specific case of accreditation as a QI effort [22]. Our results are consistent with other studies and systematic reviews [4, 6, 13, 34] about the influence of context factors on QI, pointing out the importance of the macro and microsystem context levels, both inside the organizations, well above the environment (outside) level [13, 34], as well as some interesting findings in relation to the influence of the QI team context level.

*Internal context levels are key. Particular relevance of resources and physicians commitment for QI.*

According to the MUSIQ conceptual model [11], the *Macrosystem* and *Microsystem* dimensions provide structure and functionality for the operation and provision of services in hospitals. In these context levels, services are planned, directed, coordinated, and the actions aimed to the expected quality executed. In our study, we have observed notable differences in both levels in favor of accredited hospitals. The “existence of a responsible person for quality management or director” is the only element with no difference between accredited and non-accredited hospitals. However, this may be considered a consequence of the regulatory requirements for hospitals in Colombia, in favor of the existence of quality management structures [35]. We found the largest differences, for all its five factors, at the *microsystem* level. This may indicate that the development of structures and processes for accreditation may take place and root at the context level that is closer to the patient care [36]. A recent systematic review on the influence of the context in the effectiveness of QI strategies in hospitals [34], concludes also that particular factors at microsystem level can influence the effectiveness of QI interventions. If we are to consider accreditation as a QI project, our results indicate that this level of organization in hospitals is key. In general, significant *macro* and *micro* system factors can be grouped into the following three areas: *1) prioritization of the human talent; 2) availability and use of tools; and 3) availability of resources.* It may be argued that the link and interrelations of context in these three areas result in the improvement of processes for QI, including the achievement of accreditation. The relevance of this interrelation is described in the *MesoParadigma* [9] organizational behavior theory, which is consistent with the results of our study and supports the explanation of its results. Factors such as *leadership, culture, motivation, and organizational maturity,* significantly better valued in the accredited facilities, may be a result of the training, learning and experience of *human talent*, which can be achieved through policies and strategies that ensure *stability work*, which in turn can promote factors such as the *capacity in use of methods and tools for QI.* These findings are consistent with the study and proposal by Meyer and others [37]. For these authors, the key factors to improve quality in hospitals and to promote effective strategies for QI are: *“development of a proper culture, attraction and retention of appropriate human talent, design and updating of the necessary processes and put at the disposal of employees the appropriate and necessary tools for their work”*. The results of our logistic multivariable model clearly point out to the particular importance of the *availability of resources*, in fact the most important explanatory element for the achievement of accreditation in hospitals health of medium and high complexity in Colombia, in combination with the *involvement of physicians*. Similarly, the studies by Kaplan et al. applying the MUSIQ model [13] show that the *availability of resources* have the most consistent and strong effect to achieve success in QI projects. On the same lines, the *“involvement of doctors”* has been recognized almost as a requisite for QI [38], and the definition of strategies to motivate and involve them as an essential component to achieve positive outcomes [39, 40].

The findings for the Quality team context factors, the lower internal context level, deserve a separate comment. In this level, there are fewer factors with significant differences between the two study groups, as compared to other internal context levels. The only factor at the Quality team level associated to a higher probability of being accredited is the stability of the QI team (Table 3). This suggests that for all evaluated hospitals, there are structures and processes to respond to the needs of quality management, and that what it is important is a greater organizational consolidation of these units. Other studies addressing the benefits of participation in accreditation processes, suggests that it can stimulate change [41, 42], and that the composition of the QI teams is one major determinant for the effectiveness of QI strategies [36]. However, the accreditation is an organization-wide process and probably the involvement of other structures become more important, beyond the specific QI team [43].

### The apparently contradictory influence of the environment

Apart from the *strategic decision of the board of trustees or directors*, the environment context factors do not show results in favor to accreditation in our study. Moreover, the factor *external motivation/market incentives*, hypothetically important for accreditation, is significantly felt and valued higher in the facilities that were not accredited. Similarly, the pursuit of *institutional prestige* is almost significantly more valued in non-accredited hospitals. These results can be interpreted on one hand in terms of a seemingly lesser influence of *environment* context factors as compared to internal context factors, and on the other hand because it seems that these external factors are no longer challenges once the facility get accredited. A literature review on accreditation in Canada found similar results. It was evident that after first year of accreditation and particularly after 10 years, institutions no longer find in the accreditation a challenge [44].

### Limitations

Generalization of results may be limited by sample size and the self-selection of participating hospitals, particularly in the control group of (non-accredited) hospitals, which had a lower response rate, even though cases and controls were matched by their structural characteristics. We have not assessed whether the need of a series of reminders, particularly for the control group, could affect the quality of responses, nor the possible presence of social desirability bias. We think, however that both are unlikely, given the type of questions, most of them without an apparent “good” answer. In any case, if present, social desirability bias would probably affect mostly the control group, who maybe would like to look better that they actually are. We may hypothesize then than the actual differences with the group of accredited hospitals would be even greater.

## Conclusion

This study has applied an instrument, based on the MUSIQ model, which allows assessing the relevance of different context factors and organizational levels to explain success in the accreditation process in Colombia. Our results are generally consistent with the expected according to the international literature. Internal macrosystem and microsystem seem to be more relevant than external environment factors. We encourage further studies to consolidate this model, and to contribute to the understanding of the factors and drivers leading to accreditation, as well as to any other QI related project.

## Supplementary information

**Additional file 1:** **Annex 1.** Frequency of matching variables in cases (accredited) and control (non accredited) hospitals.

**Additional file 2: Annex 2.** List of questions to assess context factors.

## Data Availability

The dataset generated and analyzed in this study is available in a Figshare repository: 10.6084/m9.figshare.7221923.v1

## Abbreviations

QI: Quality improvement
MUSIQ: Model for Understanding Success in Quality
OR: Odds ratio
REPS: Register of Providers of Healthcare Services (in Spanish)
SOGC: Mandatory System of Quality Assurance (in Spanish)
