# Supplementary material for: Contextual factors favouring success in the accreditation process in Colombian hospitals: a nationwide observational study
Source: BMC Health Serv Res. 2020 Aug 20;20:772. doi: 10.1186/s12913-020-05582-y (PMC7441620; doi:10.1186/s12913-020-05582-y)
Supplement: Supplementary file 1 — Additional file 1: Annex 1. Frequency of matching variables in cases (accredited) and control (non accredited) hospitals. [file 12913_2020_5582_MOESM1_ESM.docx]

| **Annex 1. Frequency of matching variables in cases (accredited) and control (non accredited) hospitals.** | | | | | | |
| --- | --- | --- | --- | --- | --- | --- |
|  | **Type of Ownership** | | **Complexity** | | **Recruitment of human talent** | |
|  | Private | Public | High | Medium | Own contract | By third parties |
| **Cases** | 12 (75%) | 4 (25%) | 12 (75%) | 4 (25%) | 17.260 (62%) | 10.386 (38%) |
| **Controls** | 24 (63%) | 14 (37%) | 25 (66%) | 13 (34%) | 16.924 (52%) | 15.874 (48%) |
| Total | 36 (67%) | 18 (33%) | 37 (69%) | 17 (31%) | 34.184 (57%) | 26.260 (43%) |
