# Supplementary material for: Contextual factors favouring success in the accreditation process in Colombian hospitals: a nationwide observational study
Source: BMC Health Serv Res. 2020 Aug 20;20:772. doi: 10.1186/s12913-020-05582-y (PMC7441620; doi:10.1186/s12913-020-05582-y)
Supplement: Supplementary file 2 — Additional file 2: Annex 2. List of questions to assess context factors. [file 12913_2020_5582_MOESM2_ESM.docx]

**Supplementary material: Annex 2**

**List of questions to assess context factors**

**ENVIRONMENTAL CONTEXT**

1. Pressure or market incentives for competitiveness, motivated or motivate our hospital to seek accreditation or certification of higher standards of quality in health

2. Outer organizations to the hospital (e.g. associations, Ministry of Health, national or international collaborative projects) deliver or have delivered to the hospital, advice, money, training or other resources for the development of quality projects with the objective of achieving Accreditation or Certification of higher standards of quality in health

3. The search for Institutional PRESTIGE has played a preponderant role to seek Institutional Accreditation or Certification of higher standards of quality in health in our hospital

4. The Board of Directors of the hospital has defined as a goal the achievement or maintenance of the Institutional Accreditation or the Certification of higher standards of quality in health

**MACROSYSTEM CONTEXT**

5. The Manager and senior managers of the hospital are involved in activities to improve organizational quality

6. The hospital has in its structure at the upper management level, an area and have designated a responsible for leading the quality management

7. The hospital is a dynamic and enterprising institution. People here are willing to take risks that lead to the development of the hospital

8. The manager and senior managers of the Hospital are risky and encourage employees to take risks and be innovative

9. What binds and produces union in the human team of our hospital is a commitment to innovation and development. There is an emphasis and desire to be the best

10. The hospital prioritizes the growth and acquisition of new resources. It is important to be prepared to face new challenges

11. Quality improvement is a very important issue and is integrated into all the processes, programs and projects that are developed in the hospital

12. The hospital promotes and facilitates for its staff, the development of competencies and skills to identify opportunities for improving the quality of processes and procedures

13. The staff of the Hospital is promoting and facilitating their training in the management of computer tools, statistics and indicators-based management, to facilitate their active participation in improving the quality of the hospital

14. Hospital staff are trained to perform at their job, before initiating activities and responsibilities assigned to them

15. The hospital has established a system of stimuli, incentives and recognitions that make the staff's efforts to participate in the improvement of quality in the hospital visible

16. The financial, personnel and support resources needed to implement uninterrupted quality improvement programs are valued and assigned in the hospital.

17. The information systems available in the hospital, facilitate management for quality improvement in administrative and clinical-care processes

18. The modalities of recruitment of the human resource in the hospital and the payment systems adopted for professionals and health specialists, favour the stability and low turnover of staff

**MICROSYSTEM CONTEXT**

19. In the hospitalization service it is clearly defined and recognized by all the staff and medical specialists, who leads and coordinates the service

20. Who lead or coordinate the hospitalization service, promote and engage themselves directly in activities that seek quality improvement

21. The staff of the hospitalization service considers important to improve the results in the quality of care and to modify the behaviors that may affect the safety of the patients

22. The staff of the hospitalization service knows and applies methods for quality improvement (plan-do-check-act cycle, improvement plans, causal analysis, scoreboards)

23. Who lead the hospitalization service coordinate, teach and are friendly. They help to develop all the potential of employees by becoming their guides

24. What brings together and produces union in hospital services is the emphasis on tasks and the achievement of goals. A production and quality orientation is shared within the group

25. In the hospitalization service is emphasized the quality and achievement of the proposed activities. Having measurable goals is important

26. Physicians who care for patients in the hospitalization service are actively involved in quality improvement activities

**QUALITY TEAM CONTEXT**

27. The director or quality leader of the hospital is in constant contact with the quality team of the institution and supports all actions that require its presence to lead the improvement of quality

28. The team formed to lead the quality in the hospital is composed of different professions and with diverse experience in the health sector

29. Team members who lead quality in the hospital have worked and have specific experience in the field

30. In the hospital quality team there is a complete opening to share information

31. The members of the hospital quality team have their own initiatives and implement new actions that seek to improve the quality of the hospital

32. Interventions by hospital quality team members are listened to and taken into account for their implementation

33. The important decisions taken by the hospital quality team are generally adopted by consensus of the group

34. The hospital's quality team uses techniques and methods for quality improvement (e.g. plan-do-check-act cycle, cause analysis, management and monitoring panel with indicators)

35. In general terms the team that coordinates the quality issue in the hospital has been stable and its members have worked together for a long period of time
